# Integrating Traditional Healers into the Health Care System: Challenges and Opportunities in Rural Northern Ghana

**DOI:** 10.1007/s10900-017-0398-4

**Published:** 2017-07-05

**Authors:** Eva Krah, Johannes de Kruijf, Luigi Ragno

**Affiliations:** 1 University of Dundee, Dundee, UK; 2 Department of Cultural Anthropology, Utrecht University, Padualaan 14, 3584 CH, Utrecht, the Netherlands; 3 Utrecht University, Utrecht, the Netherlands; 4 UNICEF, Accra, Ghana

**Keywords:** Traditional medicine, Ghana, Integration, Rural health care

## Abstract

Traditional medicine is widespread in Ghana, with 80% of Ghanaians relying on its methods for primary health care. This paper argues that integrating traditional and biomedical health systems expands the reach and improves outcomes of community health care. Moving beyond literature, it stresses the importance of trust-relationships between healers and biomedical staff. Insights are based on qualitative research conducted in Ghana’s Northern Region (2013–2014). Five challenges to integration emerged out of the data: a lack of understanding of traditional medicine, discrimination, high turnover of biomedical staff, declining interest in healing as a profession, and equipment scarcity. Besides challenges, opportunities for integration exist, including the extensive infrastructure of traditional medicine, openness to collaboration, and grassroots initiatives. Contemplating challenges and opportunities this paper provides recommendations for integration, including: identify/select healers, promote best practices, institute appropriate forms of appreciation/recognition of healers, provide aid and equipment, use communication campaigns to promote integration and steer attitudinal change towards healers among biomedical staff. Most crucial, we argue successful implementation of these recommendations depends on a concerted investment in relationships between healers and biomedical staff.

## Introduction

Despite progress such as the establishment of the National Health Insurance Scheme (NHIS) in 2004, Ghana’s biomedical health care system continues to face profound challenges. A primary challenge is its accessibility, particularly in smaller rural communities. Recent surveys show that 70% of the Ghanaian population lives in areas with insufficient access to biomedical healthcare [1]. Reports indicate the physician-to-population ratio in Ghana is 1:20,000 [1–3]. In northern rural regions, where this study is situated, this ratio is 1:100,000 [3].

Traditional^1^ healing is the most common alternative to biomedicine in Ghana. The traditional healer-patient ratio in rural areas is 1:200 [4]. Evidence shows 80% of the rural populations rely upon traditional medicine (TM) for primary health needs [5, 6]. This can be explained by the accessibility and affordability of TM [1, 7]. Whilst Ghanaian health insurance often proves inadequate, traditional healers usually do not charge in advance. Instead, they are compensated with a small (monetary) gift if patients indeed recover [1, 7, 8]. Furthermore, TM is often considered most effective. Also, unlike biomedicine, it is embedded in cultural and moral value systems and consistent with hegemonic traditions and (religious) beliefs [1, 2, 9].

Considering the vitality of TM in Ghana, and the challenges of biomedicine, this paper makes an argument for *integrating* traditional healing and biomedical health care, as an effective and sustainable way of expanding the reach and outcomes of health care in Ghana.^2^ It adds to the body of literature that, following the WHOs (1978) commitment to promote the inclusion and integration of traditional practitioners in national health programmes [10], assesses collaborations between traditional healing and biomedical health care across sub-Saharan Africa [2, 3, 11–19]. Studies indicate such collaborations are complex and often ineffective [12, 13, 20]. This paper assesses problems hindering integration, while foregrounding opportunities. Recommendations are provided on how to facilitate integration of traditional healers into Ghana’s formal health care system. Our core argument concerns the paramountcy of interpersonal relationships between healers and biomedical staff. For successful implementation of all recommendations, a structural and heavy investment in these relationships is indispensable. Considering parallels in health care across sub-Saharan Africa, the Ghanaian case facilitates understanding of similar cases throughout the region.

## Methods

### Setting

The principal investigator (PI) conducted 6 months of research in two areas in Ghana’s northern region (NR): Dalon (Tolon-Kumbugu district) and Walewale, the capital of West-Mamprusi. Most data was gathered in Walewale where the PI lived. Its residents are mainly Mampruli-speaking Muslims. The PI travelled almost daily to nearby villages to interview traditional healers in their homes.

### Study Design and Participants Selection

This study employs ethnographic research methods to gain an in-depth understanding of (often concealed) practices and beliefs concerning traditional healing. Investing time and effort in trust-relationships with healers and patients was crucial. To facilitate this, the secretary of the local Ghana Federation of Traditional Medicine Practitioners Associations (GHAFTRAM) served as an assistant and gate-keeper. He introduced the PI to GHAFTRAM healers through snowball sampling and functioned as a cultural advisor and interpreter. Understanding and trust built over time alleviated the healers’ concerns that the PI was trying to “steal” their herbal concoctions for profit, and enabled respectful and appropriate conduct. Similarly, a local female research assistant interpreted during interviews with predominantly (>95%) female traditional birth attendants (TBAs).

Three groups of participants were selected:

- Biomedical health care staff: midwives, nurses and nurse managers and/or hospital coordinators
- Traditional medicine practitioners (TMPs), and TBAs, collectively referred to as ‘healers’ in this study.
- Patients receiving care either at biomedical health facilities or from traditional healers.

More than 20 in-depth individual interviews were conducted with healers. Purposive sampling was used to select registered GHAFTRAM healers. Five semi-structured interviews were organized with biomedical health staff from the Walewale district hospital. All formal interviews were recorded and transcribed. Furthermore, the PI conducted fifteen unstructured/informal patient interviews. Patients were selected through convenience sampling. Informal conversations were not recorded but retrieved from hand-written notes, documented and uploaded for analysis with *NVivo*.

Finally, frequent observations were scheduled at both biomedical clinics and homes of healers, from where they attend to their patients. Observational data provided insights into practices of healers; they confirmed the impressive scale of TM and served to validate information from interviews.

Ethics approval was granted by Utrecht University. Because of low levels of literacy, verbal informed consent in local languages was obtained from all participants prior to participation.

## Findings and Discussion

### Challenges to Successful Integration

Data indicates five challenges to integration can be discerned:

#### Insufficient Knowledge of Traditional Medicine

Interview data shows biomedical healthcare workers have limited insight into local understandings of health. Often, health workers staffing rural health facilities are not natives of the area/region they serve. Many are graduates or students doing a mandatory internship. A significant number does not speak the local language. Furthermore, ‘alien’ staff is unacquainted with local cultural beliefs and practices that influence health choices. A non-native midwife in the Tolon-Kumbungu district complained that pregnant women continue to use controversial herbal medicine to induce contractions, despite active discouragement. She exclaimed: “*I don’t know how, that’s their culture but I really don’t understand it, oh if only I could understand!*”.

#### Discriminatory Approach Towards Traditional Medicine

In line with literature on biomedical perceptions of TM [14, 21], this study suggests insufficient understanding coupled with status differences (based on education) leads to discrimination of healers and their patients. During interviews, some biomedical health care workers linked TM with being underdeveloped or “backward.” Also patients mention insults and the denial of care at biomedical facilities in case of exhibiting signs of the use of TM. Such poor treatment results in reluctance to seek help. It thus complicates diagnoses and increases the risk of complications from interacting (traditional and biomedical) medication/treatment. A nurse working at a remote clinic remarks:

*They will not tell you because they know they fail us […]. you know they wait till the thing is complicated, when they come they will not tell you they went to the traditional healer, they will tell you it started like that. But through your observation you know that they did their own thing…*

#### High Turnover of Biomedical Health Staff

The turnover of staff resulting from temporary staff appointments and internships undermines the establishment of a relationship of trust between healers and biomedical health staff. Such turnover is a widely described public health problem in sub-Saharan Africa, usually related to emigration of professionals [22–25]. In Northern Ghana, internal mobility is the most pressing concern. Health care workers move from public to private health sectors or transfer from rural to urban areas because of career opportunities.

Because of turnover, relations between healers and doctors are generally superficial. This obstructs integration, despite an outspoken desire to collaborate. Of course, there are regional differences regarding the level of familiarity and collaboration. However in certain remote locations, healers are often unacquainted with the local health centre staff. This is a significant deterrent to referral rates as cross-referring and collaboration is often a person-to-person endeavor. The perceived disadvantages of a high turnover are illustrated by one TBA:

*…nurses they are not at one place forever, they do transfer them so the new people who are there now, they don’t know of us, so whenever we send a pregnant woman they ask us to stay outside before they deliver the woman. After the delivery they now call us inside. If not those who were there and they know of us, whenever we send a pregnant woman they ask us to come inside and help so that we all deliver the woman*.

#### Declining Interest of Young People to become Healers

Mastering TM requires a period of apprenticeship. Usually such enskillment takes place between father and son (or mother and daughter), as the ‘power’ to heal is thought to pass on through bloodline. Many young people interviewed during this study, expressed an unwillingness to become healers. Such declining interest endangers TM and undermines the potential of integration.

There are several reasons for this declining interest, which is particularly visible among TBAs. One reason is the emergence of educational opportunities and some professional alternatives for girls. A second reason is the loss of status and attractiveness of the profession of TBA. As a result of the advocacy of biomedical superiority, the position of TBAs has shifted from ‘expert’ to ‘unqualified’ [26]^,^ harkening back to the second challenge to integration discussed above. A third disincentive for young people to learn/practice TM is that they do not “gain” anything. Traditionally, TM is regulated by a cultural framework of “greeting and asking for favours”. Charging money for medical services is considered immoral and something that will “*spoil the medicine*” [9]. Confronted with the monetization of life and the growing necessity of cash, unpaid healers increasingly struggle to meet their daily basic needs. In fact, all the healers interviewed during this research, practice traditional medicine as a secondary occupation. They are part-time healers and depend on subsistence farming for their livelihoods, a somewhat untenable set-up, considering the workload of some of the most popular healers who attend to dozens of patients each day. As one TBA said *“That is why so many women don’t want to* [be a TBA]. *Because you struggle and you don’t gain anything*”.

#### Lack of Herbs and Equipment

All interviewed healers mentioned the lack of means to practice TM. First and foremost, acquiring herbs and plants is an increasing challenge as growing populations and expanding settlements mean healers have to travel greater distances to find what they need. Storage also presents a challenge as few bottles and boxes are available. Furthermore, TBAs state they do not have gloves, soap, or clean cloth. All equipment is scarce. The case of a famous bone-setter who attends to dozens of patients every day is illustrative. Although local biomedical health staff routinely refers patients with fractures to him, the healer does not have splints and asks his children to cut wood into splint-like pieces instead.

### Opportunities for Integration

Besides challenges, opportunities for integration do exist:

#### Traditional Medicine Infrastructure

With one traditional healer for every 200 people the traditional health care network offers great potential. The TM infrastructure, rooted in local customs and belief systems, provides easy access to basic care. TMPs and TBAs are trusted within communities. They cross-refer patients, and exchange knowledge and experience. A certain level of formalization and standardization is already in place; an example is GHAFTRAM, which unites selected healers, providing them with ID and referral cards. TM is an *existing* care infrastructure with tremendous potential that should not be disregarded.

#### Willingness to Collaborate

Despite challenges, healers and biomedical health care workers are generally willing to cooperate. Healers are eager to improve their medical knowledge and skills, welcome opportunities for communication and education, and appreciate interaction with biomedical health care providers. Interaction/networking already leads to referrals and collaboration. As one healer said: “*Before we didn’t even know each other, now we work together*”. This collaborative stance of healers reflects findings from other studies [13, 14, 27]. However, whereas literature suggests biomedical practitioners are less eager to collaborate [27], our data shows that most biomedical practitioners are willing to collaborate with certain healers (e.g. bone-setters).

The words of the nurse–manager at the Walewale district hospital are illustrative. Reflecting on “*the problem of access of the orthodox* [biomedicine],*”* he advocated collaboration:

*The orthodox have to acknowledge the fact that the traditional are working* […] *and share burden. We need to understand that they have challenges. We need to help each other. Sometimes they just need raw education on how to do things better. Then the integration would be more*. […] *And if* [patients] *are going to the bone setter...I don’t see why we should not make an effort to follow the fellow and see what he is doing there*. […] *We already know that [many people] depend on them. So we should help them to do better*.

#### Promising (Grassroots) Initiatives

Organized efforts to improve collaboration do exist. For instance, the Ghanaian NGO Association of Church-Based Development Projects (ACDEP) has distributed mobile phones to healers and TBAs in an effort to enhance communication and to improve referral rate. Notwithstanding such efforts however, grassroots initiatives are particularly promising. One example is the initiative from a local GHAFTRAM association to air a radio commercial urging healers to join their association and make their expertise publicly known; healers who joined were introduced to the public health manager of the local hospital. Healers thus actively aimed to improve health care through initiating collaboration with biomedical health workers. A second example concerns a healer who established a traditional medicine ‘information centre,’ to efficiently refer patients to suitable healers, distribute information, and sell traditional medication. The same healer was designing a digital database with names and contact numbers of local healers and TBAs, and contact details of health workers and midwives from the nearest clinics and district hospital.

A final example is the cooperation between one district hospital and a bone-setter, with mutual recognition of expertise and authority. The hospital frequently sends patients to the healer, after taking X-rays. The healer can interpret X-rays and treats. Similarly, the healer refers patients with open wounds to the hospital, where they can be given blood, a drip and pain-killers. Most afternoons, the healer visits the hospital to see patients he has referred, and to discuss the treatment of complicated cases with doctors. These efforts are grassroots initiatives with a limited budget and reach. They underscore opportunities for further and systematic integration.

## Recommendations

### Identify and Select Credible Healers for Collaborative Initiatives

The first step towards an integrated system is to invest in systematic identification of healers who, on the basis of views of clients and biomedical health workers, can be recognized as professionals with recognized competence in managing diseases/illnesses [13]. Furthermore, as for instance King suggests [14] selected healers need to be dependable, show regular patient attendance, be willing to refer/collaborate and be registered with a recognized healer’s association.

To assure that only credible healers are selected we recommend a stepwise inclusion process that includes a solid monitoring and evaluation component (see King and UNAIDS [14]). We thus propose the establishment of a monitoring and evaluation team consisting of biomedical and traditional practitioners, members of healers associations, and other experts. This team has to establish a system of certification and quality assessment and manage cyclical evaluation of care quality. Furthermore, transparent agreements concerning (medical) responsibility and liability are required. Identifying/selecting the “best” healers is difficult and we agree with King and UNAIDS [14] that this requires interaction and observation.

### Select and Promote Best Collaborative Practice

A next step is to select and promote ‘best practices’. The most promising existing initiatives should be recognized and promoted as examples of an integrated approach to health care. Furthermore, promising initiatives should be structurally supported to facilitate sustainable systemic change.

### Invest in Interpersonal Relations

Along with investments in structures and systems, successful integration depends on investments in interpersonal relationships between stakeholders. The importance of face-to-face interaction cannot be overemphasized in Ghana, where greeting is a social fundamental [8, 9]. Data indicates that all successful initiatives for collaboration/integration were driven by interpersonal relationships. Healers who personally know biomedical staff are more likely to refer patients to clinics. In fact, in cases where healers and doctors consider one another colleagues or friends, cross-referral systems flourish. Particularly in light of challenges to integration, such as unawareness and discrimination, a concerted investment in interpersonal relations is crucial for integration. In line with the arguments of Ae-Ngibisi et al. [2] and Wreford [16], we want to emphasize that for these relations to work, they should be balanced (non-hierarchical) and marked by mutual respect and an ongoing exchange of information/insights.

### Appreciation and Recognition

Appreciating the value of TM facilitates successful integration. TM is rooted in sociocultural principles of respect and reciprocity [7, 9] and healers receive respect and gratitude in return for their services. They are often rewarded with small gifts or modest amounts of money by patients. (Symbolic) tokens of appreciation are great motivators. Illustrative is the distribution of t-shirts and identification cards among healers by a local NGO. This enhanced the healers’ status and proved a strong motivator. Status-building/appreciation should be considered in integration strategies.

### Basic Supplies

Selected healers should (structurally) receive supplies and equipment. First, there is a need for basic, inexpensive equipment (e.g. gloves, soap, boxes/bottles, and splints). Furthermore, TM practitioners would benefit from running water (hygiene) and means of transportation. Finally, especially TMPs need help with the accommodation of patients. Also, establishing a fair system of reimbursement will aid integration. All these measures are cost-effective compared to investments in hospital care.

### Communication Strategy

Recommendations above should be accompanied by a communication strategy aimed at (a) publicly explaining and promoting an integrated system, (b) informing and motivating biomedical staff (c) and encouraging and training healers.

## Conclusion

Traditional healers carry much of the burden of health care in rural Ghana, and there should be a concerted effort to incorporate them into the national health care delivery system. This paper argues that traditional and biomedical health systems should be integrated, as an effective and sustainable way of expanding the reach and improving outcomes of health care in Ghana.

Findings reveal that integration is hindered by: the biomedical health workers’ lack of knowledge of traditional medicine, resulting for instance misunderstanding and discrimination; high turnover of biomedical health staff undermining instrumental relations; declining interest of young people in becoming healers; and healers’ lack of medicine and equipment. Yet, opportunities for integration do exist. Most important is the extensive infrastructure traditional care offers. Also, data show a broad willingness to collaborate. Finally, promising grassroots initiatives have surfaces, and can guide the way forward.

On the basis of the current study, we recommend identification of key healers and selection/support/promotion of the most promising grassroots initiatives. Also, there must be a pointed investment in power-sensitive relationships between healers and biomedical health workers. Government and NGO policies typically target healer-to-hospital referrals without paying enough attention to actual *dialogue* between traditional and biomedical staff. However, face-to-face communication and interpersonal contact are key to successful and sustainable change in the social context of rural Ghana. Crafting change, therefore, fundamentally depends on the systemic investment in boosting interpersonal contact between stakeholders. Healers should be appreciated and recognized, symbolically and materially, through basic aid and equipment. These efforts should be accompanied by a communication strategy aimed at explaining and promoting integration and educating stakeholders. With these recommendations, directed at fostering interpersonal relationships, trust, and mutual understanding, a sustainable system of integration can be developed whereby integration of traditional healers would result in an improved health care delivery system in rural Ghana.

## Footnotes

1 The WHO defines traditional medicine as: “the sum total of the knowledge, skill, and practices based on the theories, beliefs, and experiences indigenous to different cultures, whether explicable or not, used in the maintenance of health as well as in the prevention, diagnosis, improvement or treatment of physical and mental illness” [4].

2 The Ebola epidemic of 2014 in West Africa and its concomitant risk of spreading across the world demonstrated, improving local health care has global pay-offs. It also reveals the dangers of a lack of integration/understanding. (Foreign) medical aid workers were uninformed about local customs and belief systems, and local populations tended to seek advice and care from traditional healers whom they knew and trusted [11]. Medical interventions by the international community were thus severely hampered [12].
